# Dental pulp–derived stem cells inhibit osteoclast differentiation by secreting osteoprotegerin and deactivating AKT signalling in myeloid cells

**DOI:** 10.1111/jcmm.16071

**Published:** 2021-01-28

**Authors:** Suman Kanji, Ripon Sarkar, Asmita Pramanik, Sudhir Kshirsagar, Carl J. Greene, Hiranmoy Das

**Affiliations:** ^1^ Department of Pharmaceutical Sciences School of Pharmacy Texas Tech University Health Sciences Center Amarillo TX USA

**Keywords:** dental pulp‐derived stem cells, M2 polarization, osteoclast differentiation, osteoprotegerin, pAKT

## Abstract

Osteoclasts (OCs) differentiate from the monocyte/macrophage lineage, critically regulate bone resorption and remodelling in both homeostasis and pathology. Various immune and non‐immune cells help initiating activation of myeloid cells for differentiation, whereas hyper‐activation leads to pathogenesis, and mechanisms are yet to be completely understood. Herein, we show the efficacy of dental pulp–derived stem cells (DPSCs) in limiting RAW 264.7 cell differentiation and underlying molecular mechanism, which has the potential for future therapeutic application in bone‐related disorders. We found that DPSCs inhibit induced OC differentiation of RAW 264.7 cells when co‐cultured in a contact‐free system. DPSCs reduced expression of key OC markers, such as NFATc1, cathepsin K, TRAP, RANK and MMP‐9 assessed by quantitative RT‐PCR, Western blotting and immunofluorescence detection methods. Furthermore, quantitative RT‐PCR analysis revealed that DPSCs mediated M2 polarization of RAW 264.7 cells. To define molecular mechanisms, we found that osteoprotegerin (OPG), an OC inhibitory factor, was up‐regulated in RAW 264.7 cells in the presence of DPSCs. Moreover, DPSCs also constitutively secrete OPG that contributed in limiting OC differentiation. Finally, the addition of recombinant OPG inhibited OC differentiation in a dose‐dependent manner by reducing the expression of OC differentiation markers, NFATc1, cathepsin K, TRAP, RANK and MMP9 in RAW 264.7 cells. RNAKL and M‐CSF phosphorylate AKT and activate PI3K‐AKT signalling pathway during osteoclast differentiation. We further confirmed that OPG‐mediated inhibition of the downstream activation of PI3K‐AKT signalling pathway was similar to the DPSC co‐culture–mediated inhibition of OC differentiation. This study provides novel evidence of DPSC‐mediated inhibition of osteoclastogenesis mechanisms.

## 1 INTRODUCTION

Osteoclasts (OCs) are multinucleated cells, differentiated from the monocyte/macrophage lineages under the influence of pathological stimulations, such as soluble receptor activator for NF‐κB ligand (RANKL), that contribute to the bone resorption and remodelling. Bone remodelling is primarily regulated by interactions between bone‐forming cells, osteoblast (OB) and bone‐resorbing cells, and OCs.^1^ A precise and orchestrated molecular communication among OBs, OCs, bone cells and other bone marrow cells is necessary in regulating bone formation and resorption.^2^ OBs, stromal cells and T cells express receptor activator for NF‐κB ligand (RANKL) and secrete macrophage colony‐stimulating factor (M‐CSF). The binding of RANKL and M‐CSF to RANK and M‐CSF receptor, respectively, induces osteoclastogenesis.^3^ The main switch for osteoclast‐mediated bone resorption is the RANKL, a cytokine that is released by activated OBs. Its action on the RANK receptor is regulated by osteoprotegerin, a decoy receptor that is also derived from OBs.^2^ RANK is highly expressed on the surface of osteoclast progenitors and mature OCs, which translate osteoclastogenesis signals by binding to RANKL.^4^, ^5^ In pathological conditions, these essential signals for OC differentiation initiate from myeloid cells, specifically, inflammatory monocytes, macrophages and dendritic cells.^6^, ^7^ In normal condition, OCs are essential for skeletal morphogenesis and restructuring. However, OC‐mediated excessive resorption of bone is evident during various pathological conditions, such as arthritis, osteoporosis and Paget's bone disease.^2^, ^8^

Inflammation is a cellular defence mechanism against foreign molecules that plays a critical role in maintaining cellular response. However, sustained inflammatory responses result in the development of pathological condition such as rheumatoid arthritis (RA) that is often associated with bone and cartilage destruction. The presence of pro‐inflammatory cells and cytokines in the synovium initiates the inflammatory process, and leads to the damage of cartilage and bone, resulting in deformity of the joints and compromised quality of life. The progressive nature of bone destruction is specifically involved in activating OCs by interaction with synovial fibroblasts and immune cells.^9^, ^10^ Differentially activated macrophages play a key role in the pathophysiology of various types of cytoskeletal and other inflammation‐related diseases.^11^ During initiation of inflammation, macrophages are activated and termed as pro‐inflammatory M1 phenotype, which promote osteoclast differentiation and accelerate tissue damage by releasing high levels of pro‐inflammatory cytokines, such as TNF‐α.^9^ In contrast, the anti‐inflammatory macrophages are called as M2 phenotype, which counteract pro‐inflammatory conditions by secreting anti‐inflammatory cytokines, and scavenging cellular debris. The M2 macrophage can be induced by the cytokine interleukin‐4 (IL‐4).^12^, ^13^ In general, M1 and M2 myeloid cells are involved in initiating and resolving inflammation, respectively.^14^ Therefore, cell therapeutic strategy that modulates macrophage polarity may provide significant advantage by influencing uncontrolled osteoclast differentiation in diseases such as osteoporosis and RA.

Osteoprotegerin (OPG) is a secretory glycoprotein of the TNF receptors superfamily and is essential for osteoblast differentiation. OPG is produced in various tissues, such as bone, skin, liver, lung and stomach. OPG was initially found in human embryonic fibroblasts and was termed as osteoclastogenesis inhibitory factor (OCIF) because of its role in inhibition of osteoclast differentiation.^15^ Bone‐forming ability of OPG has recently demonstrated in ovariectomized mice where the treatment of OPG resulted in increased bone mineral density and bone volume with simultaneous reduction in active OCs.^16^ OBs negatively regulate OC differentiation and function by producing OPG. It was shown that OPG inhibits the OC differentiation and acts as a decoy receptor by sequestering RANKL.^17^ RANK‐mediated signalling pathways are also involved in TNF receptor–associated factors (TRAFs) and lead to the activation of nuclear factor кB (NF‐кB), c‐Jun N‐terminal kinase (JNK), P38, Src and AKT pathways.^18^ The activation of these signalling molecules is evident in osteoclast differentiation and bone resorption.

Dental pulp–derived stem cells (DPSCs) are mesenchymal in nature, retain self‐renewal and mutipotential capacity, and have potential for tissue regeneration.^19^, ^20^ These cells have been shown to maintain their 'stemness' without changes in either morphology or expression of stem cell markers over long period of time in culture.^21^ This multipotency, especially the ability to differentiate towards the osteogenic lineages, is attractive for the development of cell‐based therapeutics for bone and cartilage in various pathological disorders, including RA, osteoarthritis and osteoporosis.^22^, ^23^ In addition, DPSCs also have been shown to possess immunosuppressive effects,^24^ suggesting the potential use of human DPSCs for inflammatory and autoimmune diseases. Furthermore, DPSCs secrete a variety of growth factors and cytokines that employ paracrine activities on various cells for their functionality and differentiation.^25^, ^26^ In addition, DPSC shares most of the characteristics with the human mesenchymal stem/progenitor cells (MSC). MSCs isolated from human bone marrow, adipose tissues, placenta, umbilical cord and other tissues are currently being administered to large numbers of patients as an autologous and allogeneic stem cell therapy. Over 1000 clinical trials with MSC have been registered,^27^ and several have reached phase II or phase III stage of development ^28^ with minimal side effects. Human MSCs were shown to have positive effects in experimental disease models in mice without immune rejections.^29^, ^30^, ^31^, ^32^

Current investigation focuses on elucidating underlying mechanisms by which DPSCs exert its inhibitory effects on RAW 264.7 cell differentiation into OCs, which are responsible for bone resorption in various disorders of bones and cartilages.

## 2 MATERIALS AND METHODS

### 2.1 Reagents and antibodies

Monocytic/macrophage cell line (RAW 264.7) was obtained from American Type Culture Collection (ATCC^®^ TIB‐71). Soluble (s) RANKL (315‐11‐100 UG) and M‐CSF (315‐02‐100 UG) were obtained from PeproTech Incorporation. The tartrate‐resistant acid phosphatase (TRAP) assay kit (387A‐1KT) was obtained from Sigma‐Aldrich Corporation. DAPI (D1306) was purchased from Invitrogen Corporation. Antibodies for NFAT2/NFATc1 (8032) and GAPDH (2118S) were purchased from Cell Signaling Technology. Antibody for osteoprotegerin (sc‐390518) was purchased from Santa Cruz Biotechnology. The cathepsin K (ab188604), TRAP (ab185716) and MMP9 (ab38898) antibodies were purchased from Abcam.

### 2.2 Cell culture

Human dental pulp‐derived stem cells (DPSC) were isolated from discarded third molar teeth, which were obtained after surgical extraction from a healthy adolescent donor in clinic with prior approval from the Institutional Review Board (IRB), TTUHSC, Amarillo, and consent from donors and parents as applicable. Teeth were thoroughly washed (at least 3 times) with phosphate‐buffered saline (PBS) containing 100 U/mL of penicillin, 100 μg/mL streptomycin and 2.5 μg/mL of amphotericin B (antibiotic‐antimycotic solution, 15240062; Gibco, Thermo Fisher, Waltham, MA, USA). Teeth were cut open to harvest the pulp, which was then minced into approximately 1‐mm cubes and plated onto 60‐mm cell culture plates and cultured with alpha (α) modified Eagle medium (MEM, Gibco) supplemented with 20% FBS (HyClone, Thermo Fisher Scientific, USA), 2 mmol/L glutamate (Gibco) and 100 U/mL of penicillin, 100 μg/mL streptomycin and 2.5 μg/mL of amphotericin B (all from Gibco). Fresh medium was added every third day of culture after removing the old medium. Cells that migrated from the pulp tissues and became confluent were collected by dissociation by scraping or non‐enzymatic dissociation buffer (Cellgro, 25056Cl), and were re‐cultured as passage 1. Cell viability was determined using the Trypan blue exclusion method. Experiments were performed using cells between 3 and 10 passages. RAW 264.7 cells were cultured and grown in DMEM containing 10% heat‐inactivated FBS and antibiotics (100 U/mL of penicillin and 100 μg/mL streptomycin) at 37°C in a 5% CO_2_ atmosphere according to the standard protocol and used for various experiments.

### 2.3 Primary monocyte isolation

Thirty mL of fresh human (adult) peripheral blood was collected from each donor through venipuncture with the help of staff members from the Coffee Memorial Blood Institute, Amarillo, TX, with appropriate consent from the donor and with the approved IRB from the Texas Tech University Health Sciences Center. Peripheral blood mononuclear cells (PBMCs) were collected by using Ficoll‐Paque gradient centrifugation. PBMCs (5 × 10^6^) were plated on sterile coverslip placed on a well of a 6‐well cell culture plate and incubated for 2 hours at 37°C within the tissue culture incubator. Non‐adherent cells were removed by gentle washing with the cell culture medium (DMEM). Adherent cells were then subjected to the osteoclast differentiation and staining as described below.

### 2.4 Osteoclast differentiation

RAW 264.7 cells were cultured at a density of 2 × 10^5^ cells/well in 6‐well plates in DMEM containing 10% FBS and antibiotics (100 U/mL of penicillin and 100 μg/mL streptomycin) in the presence of 50 ng/mL recombinant mouse sRANKL and 20 ng/mL recombinant mouse macrophage colony‐stimulating factor (M‐CSF/CSF1).^33^ M‐CSF and sRANKL were replenished along with medium once every two days for 6 days. Cultures were maintained at 37°C in a humidified 5% CO_2_ atmosphere. On day 6, culture medium was collected for the ELISA, and cells were either harvested for total RNA or protein extraction for qPCR and Western blot (WB) assays, respectively, or fixed for TRAP and immunofluorescence staining.

### 2.5 Osteoclast differentiation in the presence of OPG

Recombinant mouse OPG (TNFRSF11B‐Fc) was purchased from BioLegend (552602, CA, USA). Various concentrations of recombinant OPG (5, 25, 50 and 100 ng/mL) were added to the M‐CSF‐ and sRANKL‐containing DMEM to evaluate the effects of OPG on osteoclast differentiation. The above‐mentioned concentrations of OPG were added while changing the M‐CSF‐ and sRANKL‐containing DMEM to the respective wells. On day 6, cells were either harvested for protein extraction for WB assays or fixed for TRAP staining.

### 2.6 Osteoclast differentiation in the presence of PI3K inhibitor

PI3K inhibitor (LY294002) was purchased from Cayman Chemical (MI, USA). Various concentrations of LY294002 (1.5, 7.5 and 15 µmol/L) and 0.01% DMSO (vehicle control) were added to the osteoclast differentiation medium containing M‐CSF and sRANKL during the process of osteoclast differentiation. The above‐mentioned concentrations of LY294002 and 0.01% DMSO were added while changing the osteoclast differentiation medium to the respective wells. On day 6, cells were fixed for TRAP staining.

### 2.7 Contact‐free co‐culture assays

A co‐culture assay with RAW 264.7 and DPSCs was performed to evaluate the effects of DPSCs on osteoclast differentiation and macrophage polarization. RAW 264.7 cells (2.5 × 10^5^) in 10% DMEM were seeded in the lower wells and human DPSCs (2.5 × 10^4^ or 2.5 × 10^3^) in the upper chamber of 6‐well Transwell plates (Corning) with 0.4 µm diameter of pore size. After 16‐18 hours of seeding, upper chambers were placed on the respective co‐culture wells and simultaneously adding 20 ng/mL M‐CSF and 50 ng/mL sRANKL‐containing medium to the respective wells. Anti‐human OPG monoclonal antibody (0.1, 1 and 2 µg/mL) or isotype control IgG1 (sc390518, from Santa Cruz Biotech, and 5415 from Cell Signaling) was added to the respective wells along with osteoclast differentiating medium. On day 6, culture medium was collected for the ELISA, and cells were either harvested for RNA or protein extraction for qPCR and WB assays, respectively, or fixed for TRAP and immunofluorescence staining. For macrophage polarization experiments, RAW 264.7 cells (2.5 × 10^5^) in 10% DMEM were seeded in the lower wells and human DPSCs (2.5 × 10^4^) were seeded in the upper chamber of a 6‐well Transwell plates (Corning) with 0.4 µm diameter of pore size (ie a co‐culture of 10:1 ratio of RAW 264.7:DPSC). Total RNA was isolated after 4, 24 and 48 hours of culture.

### 2.8 RNA extraction and real‐time PCR analysis

Total RNA was isolated from RAW 264.7 cell cultured alone, or in the presence 20 ng/mL M‐CSF and 50 ng/mL sRANKL‐containing DMEM, or from DPSC co‐culture for 6 days with RAW 264.7 cell in a Transwell system, or co‐cultured with DPSC in 10% DMEM at 4, 24 and 48 hours of culture, or human DPSCs cultured in 10% and 1% FBS‐containing DMEM for 48 and 72 hours, using TRIzol reagent (Invitrogen Corporation, 15596026). One microgram of RNA was used for the synthesis of cDNA using High‐Capacity RNA‐to‐cDNA Kit according to the manufacturer's protocols (4387406; Applied Biosystems, Thermo Fisher Scientific). Real‐time PCR amplification reactions were performed using the SYBR Green PCR Kit (Applied Biosystems, 4309155). The relative expression of each target gene was quantified by calculating Ct (threshold cycle) values and normalized by β‐actin levels. Each sample was analysed in triplicate.^34^, ^35^ Human primer was denoted as (*h*). We used the following primer sets purchased from Sigma‐Aldrich Corporation and Integrated DNA Technologies: β‐actin—5′‐GGCACCACACCTTCTACAATG‐3′ (forward) and 5′‐GGGTGTTGAAGGTCTCAAAC‐3′ (reverse); Ctsk—5′‐ATGTGAACCATGCAGTGTTGGTGG‐3′ (forward)’ and 5′‐ATGCCGCAGGCGTTGTTCTTATTC‐3′ (reverse); Nfatc1—5′‐AGATGGTGCTGTCTGGCCATAACT‐3′ (forward) and 5′‐TGCGGAAAGGTGGTATCTCAACAA‐3′ (reverse); Trap—5′‐GCCTTGTCAAGAACTTGCGACCAT‐3′ (forward) and 5′‐TTCGTTGATGTCGCACAGAGGGAT‐3′ (reverse); Rank—5′‐TAGGACGTCAGGCCAAAGGACAAA‐3′ (forward) and 5′‐AGGGCCTACTGCCTAAGTGTGTTT‐3′ (reverse); Mmp9—5′‐TGAACAAGGTGGACCATGAGGTGA‐3′ (forward) and 5′‐TAGAGACTTGCACTGCACGGTTGA‐3′ (reverse); Tnf‐α—5′‐ATTCACTGGAGCCTCGAA‐3′ (forward) and 5′‐TGCACCTCAGGGAAGAATCTGGAA‐3′ (reverse); IL‐4Rα—5′‐CAAGCTCTGACCTCTGGATTA‐3′ (forward) and 5′‐AATGATGGGAGCGGGTATAAG‐3′ (reverse); Arginase 1—5′‐CCAGGGACTGACTACCTTAAAC‐3′ (forward) and 5′‐GAAGGCGTTTGCTTAGTTCTG‐3′ (reverse); Ym1—5′‐GCTAAGGACAGGCCAATAGAA‐3′ (forward) and 5′‐GCATTCCAGCAAAGGCATAG‐3′ (reverse); OPG 5′‐GCCGAGAGTGTAGAGAGGATAA‐3′ (forward) and 5′‐CTTCACCATTTCCTGGTCTCTG‐3′ (reverse); *h* OPG—5′‐CATTCTTCAGGTTTGCTGTTCC‐3′ (forward) and 5′‐CTCTCTACACTCTCTGCGTTTAC‐3′ (reverse); and *h* GAPDH 5′‐CCCTTCATTGACCTCAACTACA‐3′ (forward) and 5′‐ATGACAAGCTTCCCGTTCTC‐3′ (reverse).

### 2.9 TRAP staining

Differentiated OCs were determined by tartrate‐resistant acid phosphatase (TRAP) Assay Kit (387A‐1KT, Sigma‐Aldrich) following the manufacturer's protocol. Briefly, RAW 264.7 cells were cultured on the coverslips in a 6‐well plate for differentiation into OCs in the presence or absence of DPSC, or OPG or LY294002. On day 6 of the culture, coverslip was removed from plate and cells were fixed with 4% paraformaldehyde in PBS for 20 minutes at room temperature and then washed with PBS. Next, the mixture of solution was prepared by using sodium nitrite, Fast Garnet GBC Base solution, acetate solution, naphthol AS‐BI phosphate solution, tartrate solution and deionized water (pre‐warmed to 37°C) according to the manufacturer's protocol. This solution was added to each of the coverslips and incubated for 1 hour at 37°C in water bath protected from light. Finally, the coverslips were rinsed with deionized water thoroughly, mounted on a glass slide and examined under a light microscope (Olympus Corporation of the Americas, Waltham, MA, ix81). TRAP‐positive cells (purple) containing at least three nuclei were counted as an osteoclast cell.

### 2.10 Western blot analysis

Western blot (WB) was performed to determine the levels of NFATc1, MMP9, cathepsin K, OPG, pAKT (Ser 473) and AKT keeping GAPDH as an internal control in RAW 264.7 cells during the course of differentiation in the presence or absence of various concentration of DPSCs or OPG. The cells were lysed in 100 μL pre‐cooled RIPA lysis buffer (Millipore, Sigma‐Aldrich Corporation, 20‐188) for 30 minutes on ice and centrifuged at 12 000 *g* for 10 minutes. The supernatant was collected, and protein concentrations were estimated with Bradford's reagent (Bio‐Rad Incorporation, 5000006) using bovine serum albumin (BSA) (Sigma‐Aldrich Corporation, A7906‐10G) as a standard. Equal amounts of protein (40 μg) were separated by SDS‐PAGE gels electrophoretically and transferred to polyvinylidene difluoride membrane (Bio‐Rad Incorporation, 1620115). After blocking with 5% BSA for 1 hour at room temperature, the membranes were probed with primary antibodies for 12‐16 hour at 4°C. Then, membranes were incubated with appropriate HRP (horseradish peroxidase)‐labelled secondary antibodies (Cell Signaling Technology, 7074, and 7076) for 2 hour at room temperature. Immunoreactive protein bands were visualized by enhanced chemiluminescence (ECL, Amersham Pharmacia Biotechnology, RPN2232), and the band detections were kept within the linear range.

### 2.11 Immunofluorescence staining

To determine the protein expressions after osteoclast differentiation in the presence or absence of DPSCs, immunofluorescence analysis was performed for NFATc1, MMP9, cathepsin K and TRAP proteins. In brief, RAW 264.7 cells were grown on sterile coverslips inserted into a 6‐well plate. After 18 hour of culture, cells were induced to osteoclast differentiation with M‐CSF and sRANKL in the presence or absence of DPSC in a Transwell culture system. After 6 days of differentiation, cells were fixed with 4% paraformaldehyde (Santa Cruz Biotechnology, sc‐281692) for 30 minutes. After washing with PBS, cells were permeabilized with 0.1% Triton X‐100 (Sigma‐Aldrich Corporation, T8787) and blocked with 1% BSA. Then, cells were incubated with 200 μL of primary antibody (1:200) overnight at 4°C. After washing with PBS, cells were incubated with 200 μL of secondary anti‐rabbit or anti‐mouse antibodies (Alexa Fluor 488, A11001, or Alexa Fluor 594, A21235; 1:2000 dilution; Invitrogen Corporation) for 45 minutes in the dark. After incubation, cells were washed thrice with PBS (Gibco, Thermo Fisher Scientific, 70013‐032) and mounted with 4, 6‐diamidino‐2‐phenylindole dihydrochloride (DAPI, Invitrogen Corporation, D1306) on glass slides and sealed with transparent nail poliish. Slides were viewed under fluorescence microscope, and images were captured digitally using an Olympus ix81 microscope with SlideBook 5.0x64 software. Quantification of fluorescence stain was performed by measuring area covered by the green fluorescence intensity using ImageJ software and presented as a per cent (%) of total area.

### 2.12 ELISAs

The levels of secreted OPG were determined using human OPG enzyme‐linked immunosorbent assay (ELISA) kit in accordance with the manufacturer's recommended protocols (ELH‐OPG‐1, Ray Biotech, USA). To quantify OPG secretion from DPSCs, medium was collected from different experimental conditions, such as DPSC cultured in 10% FBS in DMEM and 1% FBS in DMEM for 48 or 72 hours; DPSCs cultured in 1 x PBS for 3, 6 or 24 hours; DPSC cultured in M‐CSF and sRANKL‐containing DMEM; and medium collected at various time‐point of osteoclast differentiation in the presence or absence of DPSCs, whereas 10% DMEM, 1% DMEM and 1 x PBS medium only without cells were used as a control in respective experiments as applicable.

### 2.13 Statistical analysis

All experiments were performed at least 3 times in triplicate, and the results were displayed as mean ± SEM. Statistical significance was ascertained by Student's t test, and *P* values less than 0.05 were considered significant.

## 3 RESULTS

### 3.1 Effect of DPSCs on osteoclast differentiation of RAW 264.7 cells

**Figure 1:**
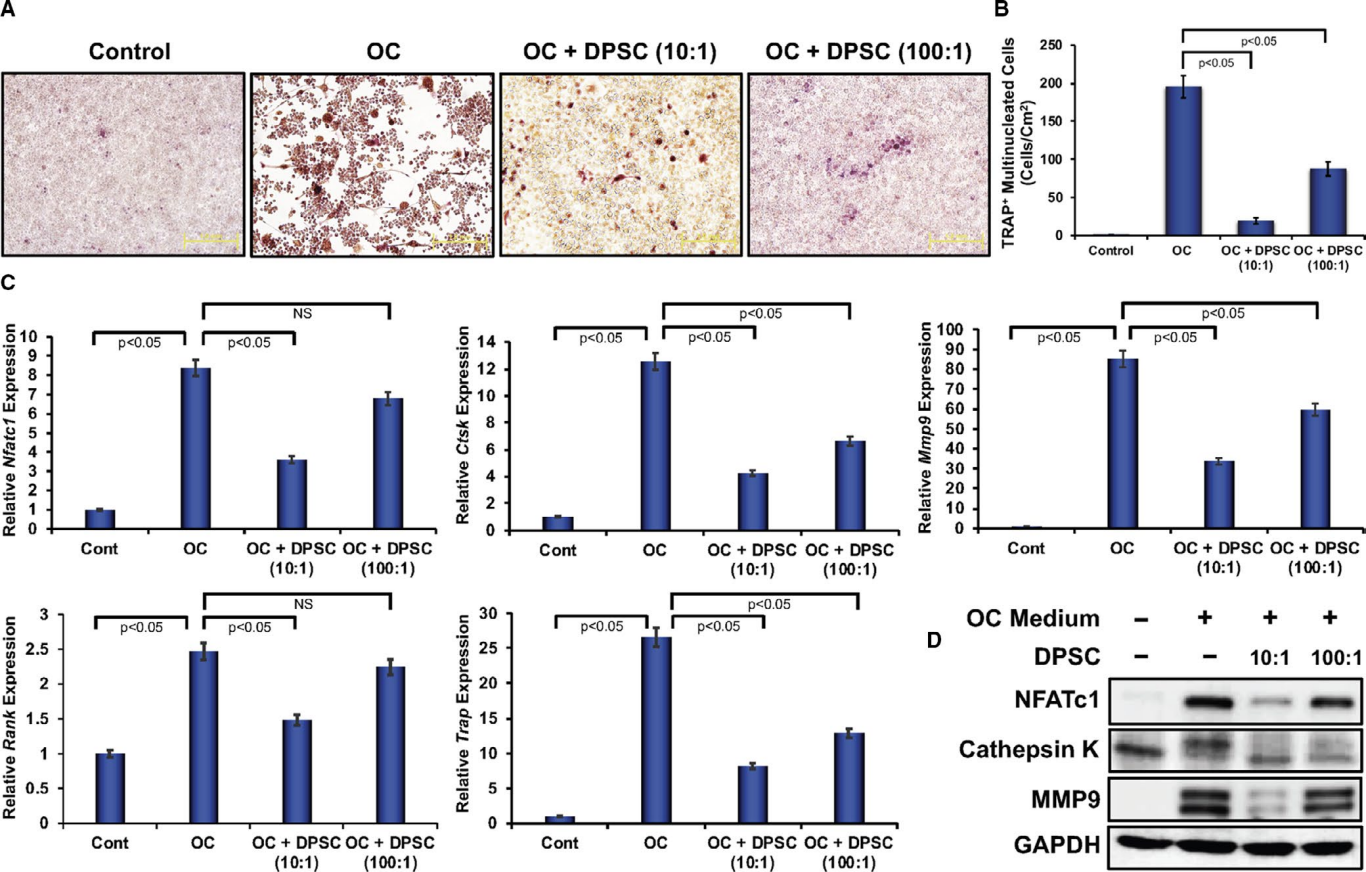
Dose‐dependent inhibition of osteoclast differentiation and related molecules by DPSCs. A, Images of induced differentiated RAW 264.7 cells determined by TRAP staining at day 6 in the absence or presence of DPSCs (contact‐free co‐culture) during osteoclast differentiation. Scale bar = 1.0 mm. B, The number of TRAP‐positive multinucleated OCs present in each group shown graphically in the absence or presence of DPSCs (contact‐free co‐culture) during osteoclast differentiation. C, Quantitative real‐time PCR analysis of osteoclast differentiation‐related marker genes such as *Nfatc1*, *Ctsk, Rank, Trap* and *Mmp9* expressions was shown graphically at day 6 in the absence or presence of DPSCs (contact‐free co‐culture) during osteoclast differentiation of RAW 264.7 cells. In all cases, *Gapdh* expressions were kept as internal controls. D, Western blotting of osteoclast differentiation‐related molecules such as NFATc1, cathepsin K and MMP9 protein levels, keeping GAPDH as an internal control, was shown at day 6 in the absence or presence of DPSCs (contact‐free co‐culture) during osteoclast differentiation

To investigate the effect of DPSCs on osteoclast differentiation, the osteoclast precursor cells (RAW 264.7) were cultured with or without DPSCs under contact‐free co‐culture system. Simultaneously, cell culture medium was supplemented with M‐CSF and sRANKL to induce osteoclast differentiation. After 6 days of induction, TRAP staining was performed to evaluate the morphology of osteoclast cells. The morphology of OCs was confirmed by multinucleated TRAP‐positive cells observed under brightfield microscope. TRAP staining revealed that multinucleated osteoclast cells were observed in cell culture plates that were stimulated with M‐CSF and sRANKL, labelled as OC (Figure 1A), whereas RAW 264.7 cells cultured without M‐CSF and sRANKL, labelled as control RAW 264.7 cells, did not show any multinucleated TRAP‐positive cells (Figure 1A). However, the abundance of multinucleated TRAP‐positive cells at the same time‐point was markedly decreased in the presence of DPSCs, which were cultured under the same osteoclast stimulants, M‐CSF and sRANKL (Figure 1A). Similar observations were also noted in experiments using human primary monocytes and human DPSCs (Figure S1A). A dose‐dependent reduction in the abundance of osteoclast cells was observed when cultured at 1:10 ratio of DPSC:RAW 264.7 cells that inhibited more effectively than that of 1:100 ratio of DPSC:RAW 264.7 cells. The number of TRAP‐positive multinucleated cells was found significantly lower in RAW 264.7 cells that were differentiated in the presence of DPSCs in contact‐free co‐culture condition compared with RAW 264.7 cells that were differentiated in the absence of DPSC (Figure 1B). Similar results were also found using human primary monocytes and human DPSCs (Figure S1B). These observations indicate that the DPSCs are capable of inhibiting osteoclast differentiation.

### 3.2 Effect of DPSCs on the expressions of osteoclast‐related marker molecules

**Figure 2:**
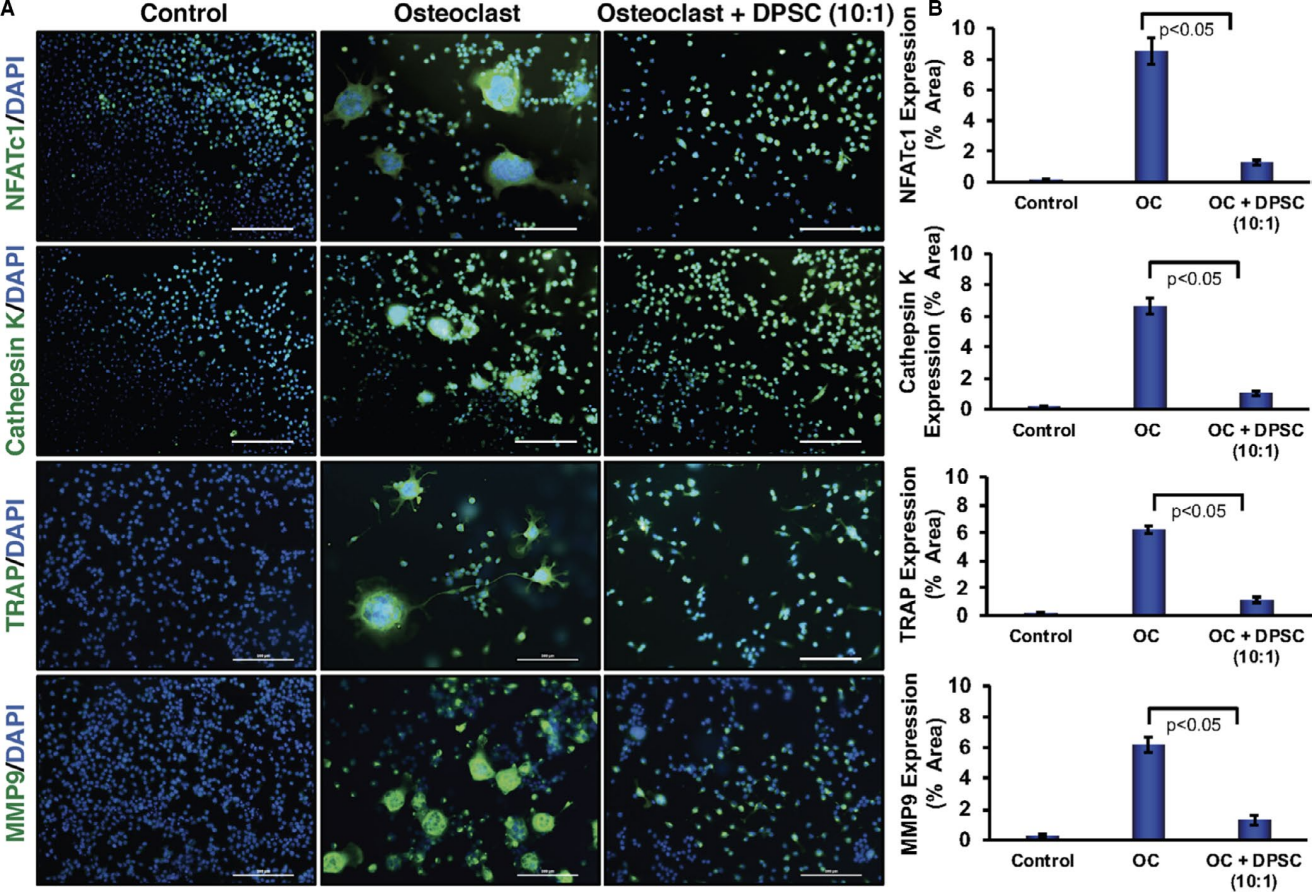
DPSCs inhibited osteoclast differentiation‐related molecules in RAW 264.7 cells. A, Immunocytochemical staining images of osteoclast differentiation determined for NFATc1, cathepsin K, TRAP or MMP9 molecules at day 6 in the absence or presence of DPSCs (contact‐free co‐culture) during osteoclast differentiation of RAW 264.7 cells. Scale bar = 500 μm. B, An amount of NFATc1, cathepsin K, TRAP and MMP9 stains were quantified for each sample shown graphically in the absence or presence of DPSCs (contact‐free co‐culture) during OC differentiation. All values are represented as mean ± SEM of 5 to 6 samples from one of the three independent experiments

To further validate the effect of DPSCs on osteoclast differentiation, we evaluated the mRNA expressions of various osteoclast‐related marker genes in RAW 264.7 cells after 6 days of induced differentiation in the presence or absence of contact‐free DPSCs. Quantitative PCR analysis revealed that key osteoclast‐related genes, such as NFATc1 (*Nfatc1*), cathepsin K (*Ctsk*), *Rank*, *Trap* and MMP9 (*Mmp9*), were significantly up‐regulated in differentiated osteoclast cells compared with undifferentiated cells (Figure 1C). These results confirm the successful differentiation of RAW 264.7 cells into OCs. However, mRNA expressions of osteoclast‐related marker genes, such as *Nfatc1*, *Ctsk, Rank, Trap and Mmp9,* were significantly decreased in differentiated osteoclast cells in the presence of DPSCs in a dose‐dependent manner, compared with respective differentiated cells (without DPSC, Figure 1C). To confirm the translation of the gene expressions to proteins, Western blot analysis was also performed from isolated total proteins after 6 days of differentiation. Western blot analysis revealed that protein levels of NFATc1, cathepsin K, MMP9 and p65 were significantly increased in osteoclast‐differentiated cells compared with undifferentiated cells. However, protein levels were markedly decreased when induced differentiation was performed in the presence of DPSCs in a dose‐dependent manner (Figure 1D, and Figure S3). Further, immunocytochemical staining confirmed that NFATc1, cathepsin K, MMP9 and TRAP‐positive multinucleated OCs were markedly increased in differentiated cells compared with control RAW 264.7 cells (Figure 2A,B), whereas a significant decreased amount of differentiation‐related proteins were observed in the presence of DPSCs (Figure 2A,B). Similar results were also found in experiments using human primary monocytes and human DPSCs (Figure S2A,B).

### 3.3 Effect of DPSCs on expressions of pro‐inflammatory and anti‐inflammatory genes in RAW 264.7 cells

**Figure 3:**
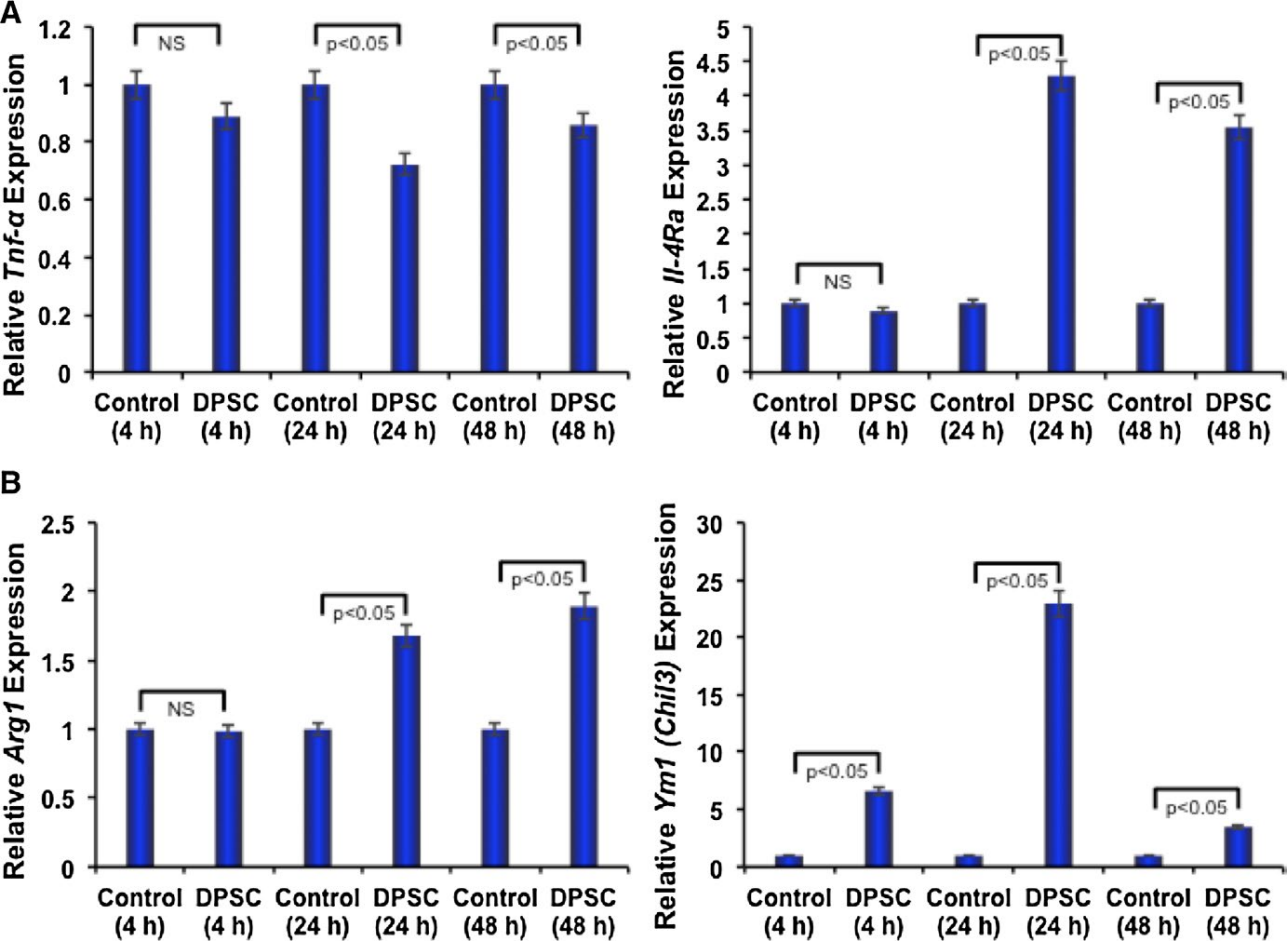
DPSCs inhibited the expression of pro‐inflammatory and induced anti‐inflammatory genes along with M2 phenotype molecules. A, Quantitative real‐time RT‐PCR analysis of pro‐ and anti‐inflammatory marker genes such as *Tnf‐*α and *IL‐4Ra,* respectively, expressions was shown graphically in the absence or presence of DPSCs (contact‐free co‐culture) in RAW 264.7 cells. B, Quantitative real‐time RT‐PCR analysis of M2 polarization marker genes such as *Arg1* and *Ym1 (Chil3)* expressions was shown graphically in the absence or presence of DPSCs (contact‐free co‐culture) in RAW 264.7 cells. In both cases, *Gapdh* expressions were kept as internal controls

DPSCs play immune modulatory role and may further regulate the osteoclast differentiation process. Hence, we wanted to test whether DPSCs have any immune modulatory role in RAW 264.7 cells. To test that, we have performed contact‐free co‐culture of RAW 264.7 cells in the presence or absence of DPSCs. Quantitative RT‐PCR analysis of RNA harvested from RAW 264.7 cells at various time‐points showed that the expressions of TNF‐α, one of the inflammatory genes, were significantly down‐regulated in the presence of DPSCs compared with control (Figure 3A). Conversely, mRNA expressions of IL‐4Rα, an anti‐inflammatory gene, were up‐regulated in RAW 264.7 cells in the presence of DPSCs compared with control (Figure 3A).

In addition, Arginase 1 (*Arg1*), Ym1 (*Chil3*), which are characteristics of M2 phenotype of RAW 264.7 cells, were significantly increased in RAW 264.7 cells in the presence of DPSCs compared with controls (Figure 3B). These data suggest that DPSCs may have the ability to polarize macrophages towards the anti‐inflammatory phenotype, which negatively regulate the osteoclast differentiation.

### 3.4 Effect of DPSCs on osteoprotegerin (OPG) expressions and secretions

**Figure 4:**
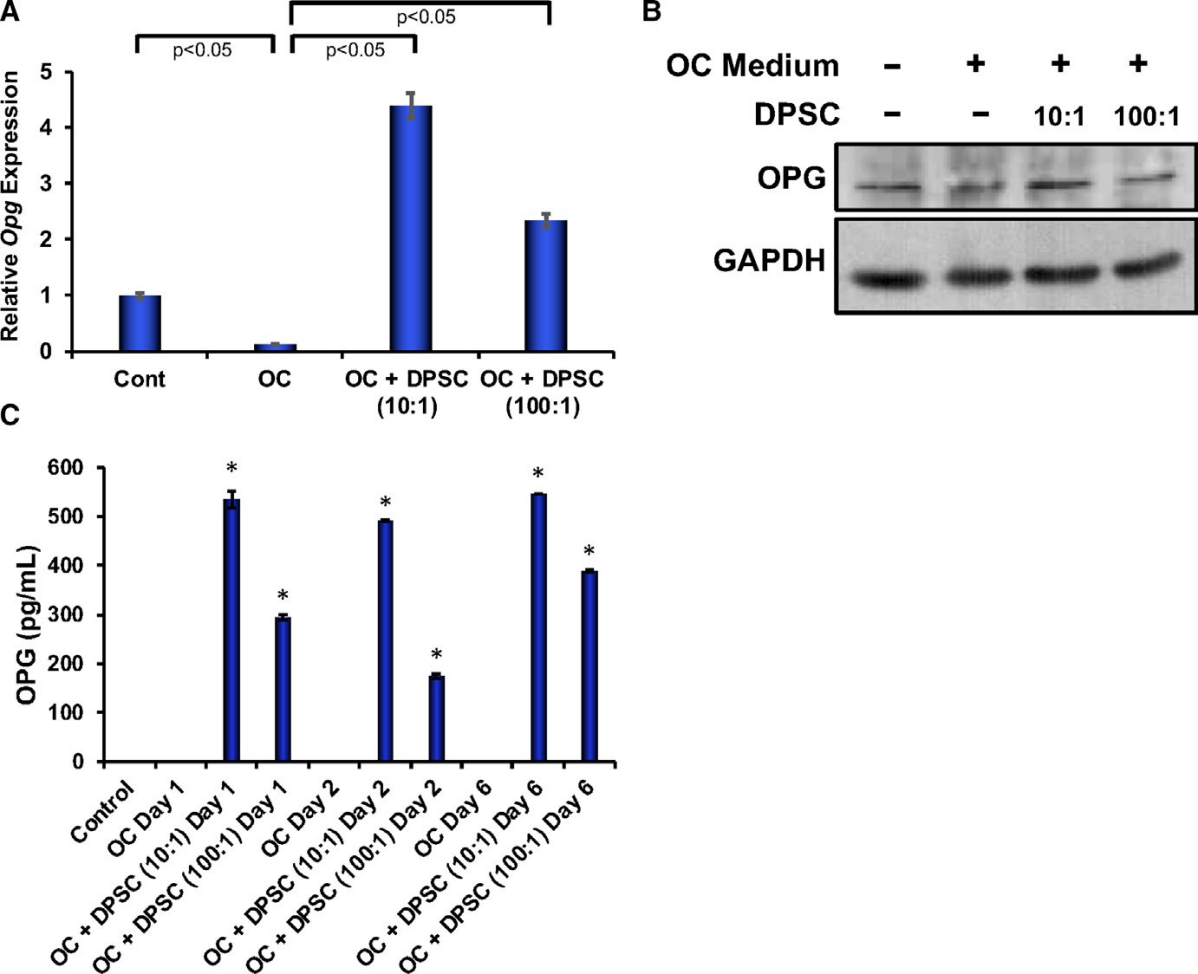
DPSC secreted OPG during osteoclast differentiation and induced OPG expression in osteoclast precursor cells. RAW 264.7 cells were co‐cultured with two different concentrations of DPSCs, or without DPSCs (assigned as osteoclast, OC) in osteoclast induction medium (sRANKL, M‐CSF and 10% FBS‐containing DMEM) using a Transwell culture system, or cultured alone in 10% FBS DMEM assigned as control RAW 264.7 cells. Total RNA and proteins were isolated after 6 d of culture from the RAW 264.7 cells, and supernatants were collected at different time‐points during the course of differentiation. A, The mRNA expression of OPG was determined by real‐time PCR from cultured RAW 264.7 cells keeping β‐actin as internal controls. B, Western blot analyses of OPG protein level shown at day 6 in the absence or presence of various concentrations of DPSC during osteoclast differentiation of RAW 264.7 cells. C, Secreted OPG in culture supernatants was measured by ELISA collected at different time‐points (after 1, 2 and 6 d of culture) during the course of osteoclast differentiation. *P < 0.05 when compared with OC of respective days. All values are represented as mean ± SEM of triplicate samples from one of the three independent experiments

To further understand the mechanism by which DPSCs are regulating myeloid cell differentiation, we evaluated the gene and protein expression of osteoclast inhibitory factor, osteoprotegerin (OPG), in RAW 264.7 cells after 6 days of induced differentiation in the presence or absence of DPSC in contact‐free co‐culture condition. Quantitative PCR analysis showed that OPG mRNA was significantly decreased in RAW 264.7 cells after 6 days of induced osteoclast differentiation compared with RAW 264.7 cells. However, when RAW 264.7 cells were induced to differentiate into OCs in the presence of DPSCs, mRNA expression of OPG was significantly increased in a dose‐dependent manner (Figure 4A). This observation was further confirmed by Western blot analysis using isolated proteins from RAW 264.7 cells from similar experimental designs. We found that OPG protein expression was increased in RAW 264.7 cells in the presence of contact‐free DPSC co‐cultures compared with differentiated OCs (Figure 4B, and Figure S4). Next, we determined which cells secrete OPG during OC differentiation using a Transwell co‐culture system. Culture supernatants were measured by ELISAs and found that there was no detectable range of OPG in supernatants collected from control RAW 264.7 cells or differentiated OCs. However, the level of OPG was significantly higher in culture supernatant in the presence of DPSC throughout the course of differentiation (Figure 4C). These results indicate that RAW 264.7 cells might not be secreting OPGs.

### 3.5 DPSCs express and secrete OPG inherently and under stressful conditions

**Figure 5:**
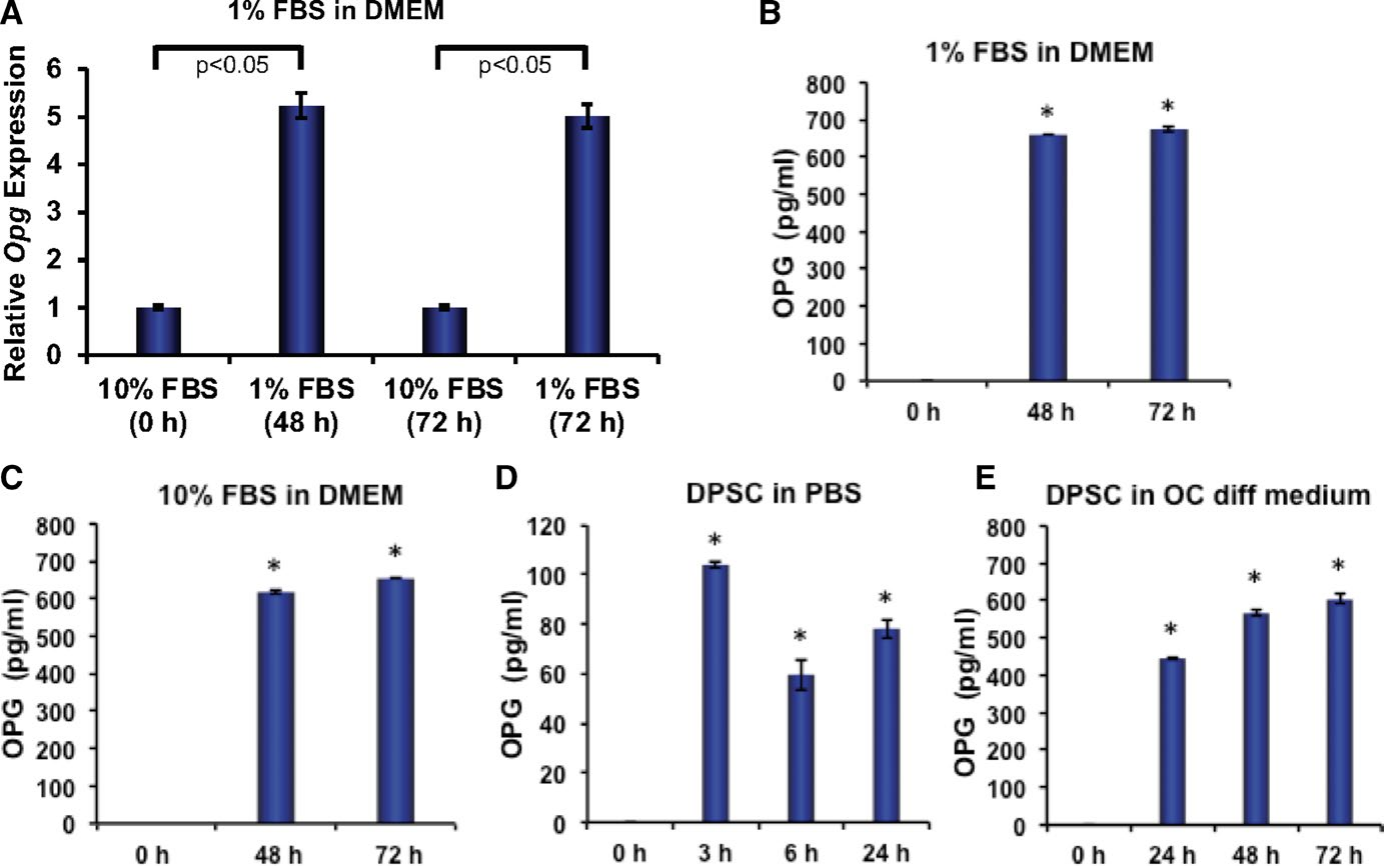
DPSC constitutively express and secrete osteoprotegerin (OPG). DPSCs were cultured in different conditions (1% FBS in DMEM, 10% FBS in DMEM, osteoclast‐stimulating media or 1 × PBS) for various time‐points (0, 3, 6, 24, 48 or 72 h), and supernatants were collected either for quantification of OPG, or cells were harvested for total RNA isolation. A, The mRNA expression of OPG was determined by real‐time PCR from cultured DPSCs keeping β‐actin as internal controls. B‐E, Quantification of secreted OPG in DPSC culture supernatants collected at various time‐points from different culture conditions. B, In 1% FBS‐containing DMEM. C, In 10% FBS‐containing DMEM. D. In 1 x PBS. E, In osteoclast differentiating medium. *= *P* < 0.05 when compared with OC. All values are represented as mean ± SEM of triplicate samples from one of the three independent experiments

To determine whether DPSC can express and secrete constitutively DPSCs was cultured under various culture conditions. In a serum starvation condition (1% FBS‐containing DMEM), cells were harvested and supernatants were collected after 48 and 72 hours of culture. The mRNA expression of OPG revealed that serum starvation significantly induced OPG expression in DPSCs in both time‐points compared with DPSCs cultured with 10% FBS‐containing medium, considered as control (Figure 5A). We further measured the amount of secreted OPG in supernatants and found that significantly higher levels of OPG after serum starvation (Figure 5B). To investigate whether DPSC secrete OPG in 10% FBS‐containing medium, we found that DPSC indeed constitutively secreted OPG in complete medium (Figure 5C). We further investigated whether DPSC can secrete OPG in compromised environment using PBS and osteoclast differentiation medium. Results indicate that in both conditions DPSCs secreted OPG (Figure 5D,E); however, the levels of OPG were lower in PBS compared with other condition, possibly because of the decreased cellular survivability in PBS. These observations indicate that DPSCs can secrete OPG constitutively and under stressed conditions, and culture medium did not have significant effect on OPG secretion.

### 3.6 Effect of OPG on osteoclast differentiation

**Figure 6:**
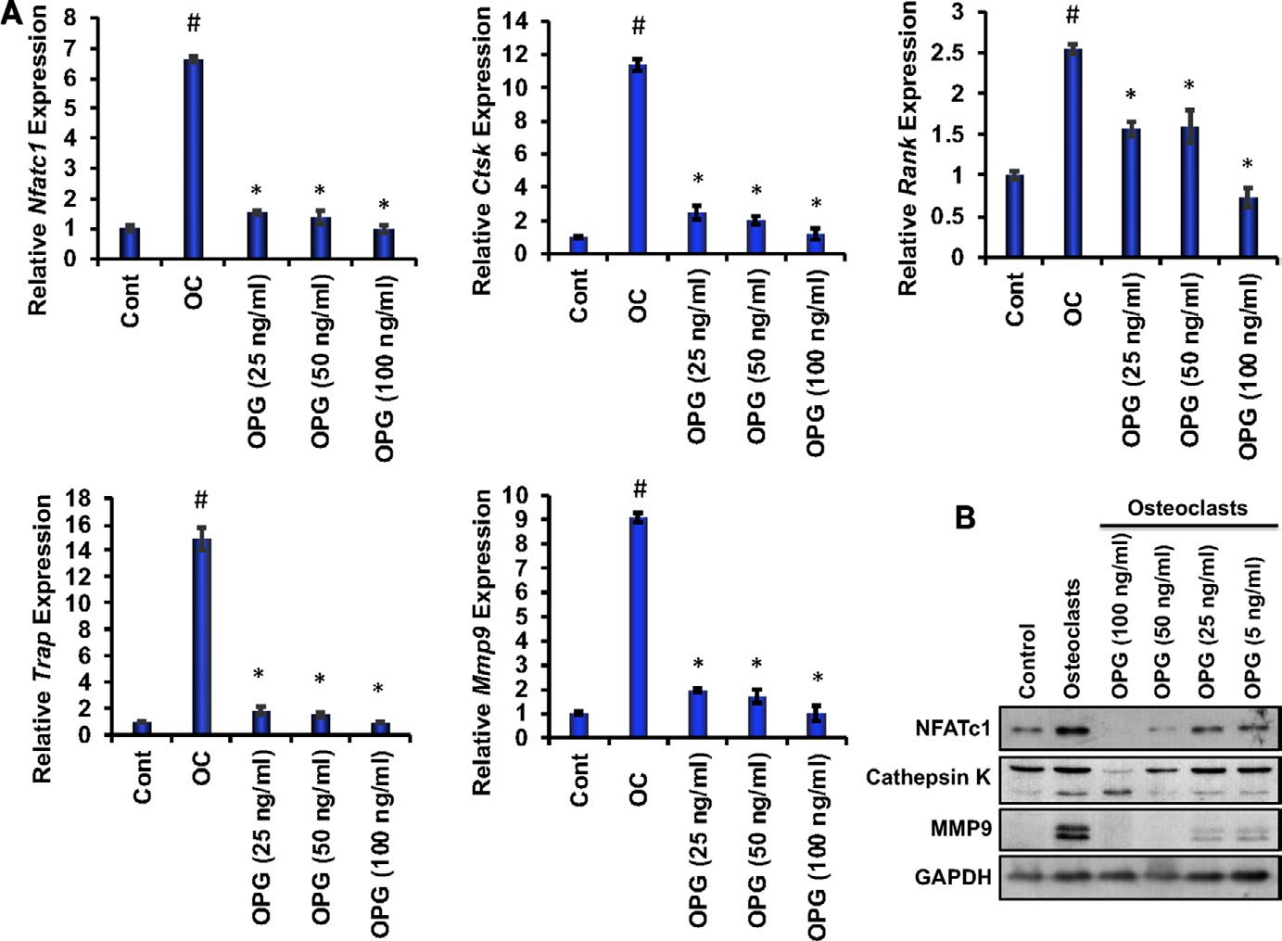
Recombinant OPG inhibited osteoclast differentiation–related molecules in a dose‐dependent manner. A, Quantitative real‐time RT‐PCR analysis of *Nfatc1*, *Ctsk, Rank, Trap and Mmp9* expression was shown graphically at day 6 in the absence or presence of various concentrations of recombinant OPG during osteoclast differentiation of RAW 264.7 cells (Cont). In all cases, *Gapdh* expression was kept as internal controls. *Gapdh* expression was kept as internal controls in all quantitative RT‐PCR analyses. (# indicates *P* < 0.05 when compared between control and day 6 of osteoclast‐differentiated samples, * indicates *P* < 0.05 when compared between control and day 6 of osteoclast‐differentiated samples in the absence and presence of either of the concentrations of recombinant OPG.) B, Western blot analyses of NFATc1, cathepsin K and MMP9 molecules shown at day 6 in the absence or presence of various concentrations of OPG during osteoclast differentiation of RAW 264.7 cells

To investigate the effects of OPG on osteoclast differentiation‐related molecules, we induced osteoclast differentiation of RAW 264.7 cells in the absence or presence of various concentrations of recombinant OPG, and real‐time RT‐PCR and Western blot analyses were performed. Quantitative RT‐PCR analysis revealed that key osteoclast‐related genes, such as *Nfatc1*, *Ctsk, Rank, Trap and Mmp9,* were significantly reduced in the presence of OPG compared with non‐treated cells after 6 days of osteoclast differentiation (Figure 6A). Further, Western blot analysis confirmed that key osteoclast‐related proteins NFATc1, cathepsin K and MMP9 levels were decreased in the presence of OPG compared with differentiated OCs (Figure 6B and Figure S5). These observations support the ability of OPG in inhibiting osteoclast differentiation‐related marker molecules.

### 3.7 DPSCs in mimicking OPG‐mediated signalling pathways of osteoclast differentiation

**Figure 7:**
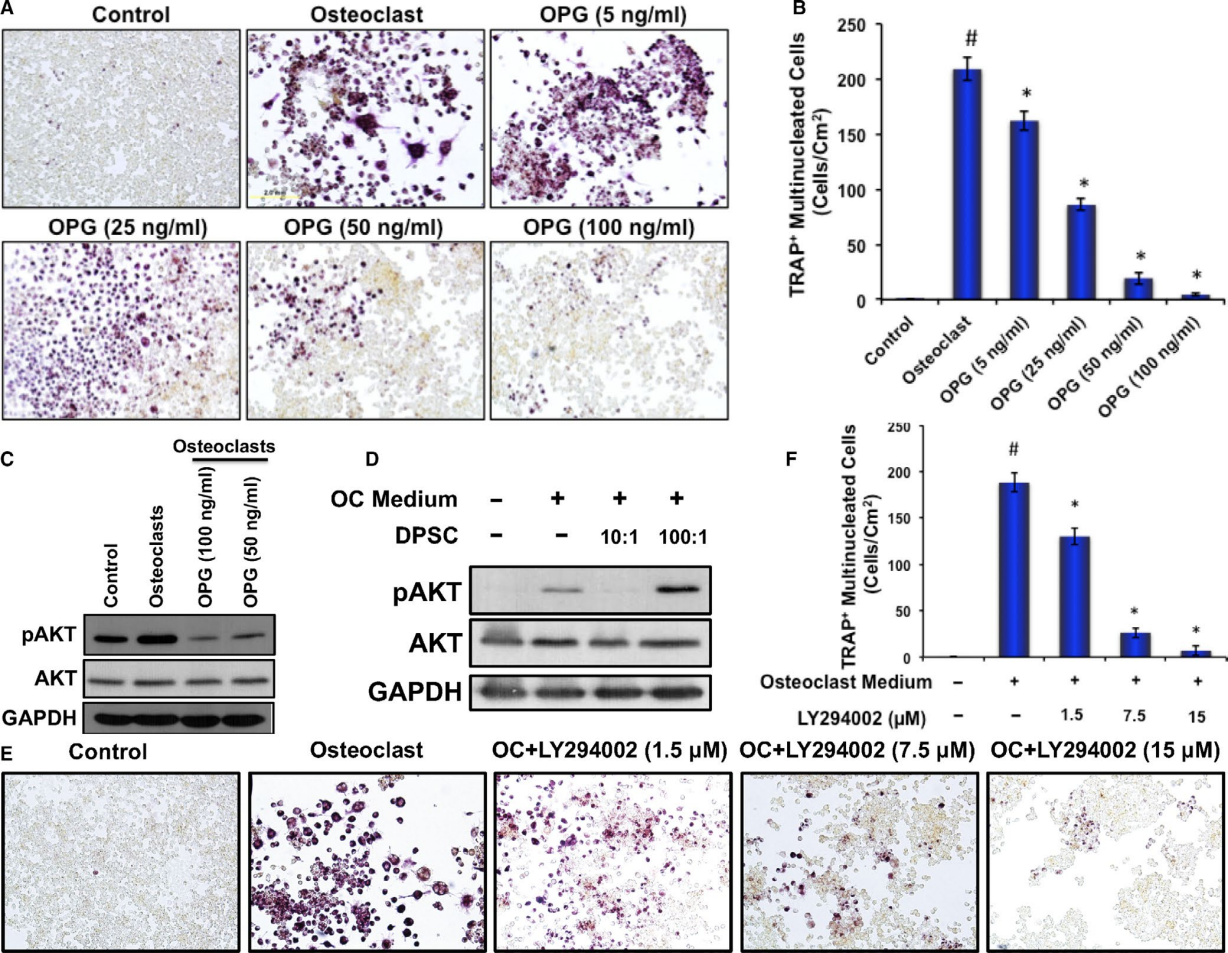
DPSC mimicked the OPG‐mediated signalling pathway (PI3K) to inhibit osteoclast differentiation. A, TRAP staining images of RAW 264.7 cells at day 6 of osteoclast differentiation in the absence or presence of various concentrations of OPG during osteoclast differentiation of RAW 264.7 cells. B, The number of TRAP‐positive multinucleated OCs present in each group shown graphically in the absence or presence of various concentration of OPG during osteoclast differentiation. C, Western blot analyses of pAKT and total AKT molecules from PI3K pathway shown at day 6 in the absence or presence of two different concentrations of OPG during osteoclast differentiation of RAW 264.7 cells. D, Western blot analyses of pAKT and total AKT molecules shown at day 6 in the absence or presence of two different concentrations of DPSCs (contact‐free co‐culture) during osteoclast differentiation of RAW 264.7 cells. E, TRAP staining images of RAW 264.7 cells at day 6 of osteoclast differentiation in the absence or presence of various concentrations of PI3K inhibitor (LY294002) during osteoclast differentiation of RAW 264.7 cells. F, The number of TRAP‐positive multinucleated OCs present in each group shown graphically in the absence or presence of various concentration of PI3K inhibitor (LY294002) during osteoclast differentiation. Scale bar = 2.0 mm

To determine the molecular mechanism of DPSC‐mediated inhibition of osteoclast differentiation of myeloid cells, RAW 264.7 cells were induced to differentiate in the absence or presence of various concentrations of recombinant OPG, or PI3K inhibitors or DPSCs. First, TRAP staining was performed after 6 days of differentiation and revealed that a concentration‐dependent effects of OPG in inhibition of osteoclast differentiation. Higher concentrations (50 ng/mL and 100 ng/mL) of OPG significantly inhibited the differentiation of OCs, whereas lower concentrations (5 ng/mL and 25 ng/mL) of OPG were not much effective in reducing osteoclast differentiation (Figure 7A,B).

To identify the regulation of key signalling molecules involved in osteoclast differentiation, total proteins were isolated after 6 days from RAW 264.7 cells induced for OC differentiation in the presence or absence DPSC or OPG as described earlier and subjected to Western blot analysis. Western blot analysis showed that activated AKT (phosphorylated AKT) was up‐regulated in the OCs; however, the addition of OPG inhibited the activation of AKT in a concentration‐dependent manner (Figure 7C and Figure S6). Inhibition of AKT activation (phosphorylation) was also observed in the presence of DPSCs in a contact‐free co‐culture (Figure 7D and Figure S7). However, total AKT level was mostly unaltered. There was no significant difference in MAP kinase molecules (P38, ERK1/2) tested in these samples (data not shown). These data indicated that DPSC‐mediated inhibition of osteoclast differentiation mimicked the signalling cascade of OPG‐mediated inhibition of osteoclast differentiation. We further confirmed the involvement of PI3K‐AKT pathways during osteoclast differentiation by using PI3K‐specific inhibitor. We found that PI3K inhibitor significantly reduced osteoclast differentiation in a dose‐dependent manner (Figure 7E,F).

**Figure 8:**
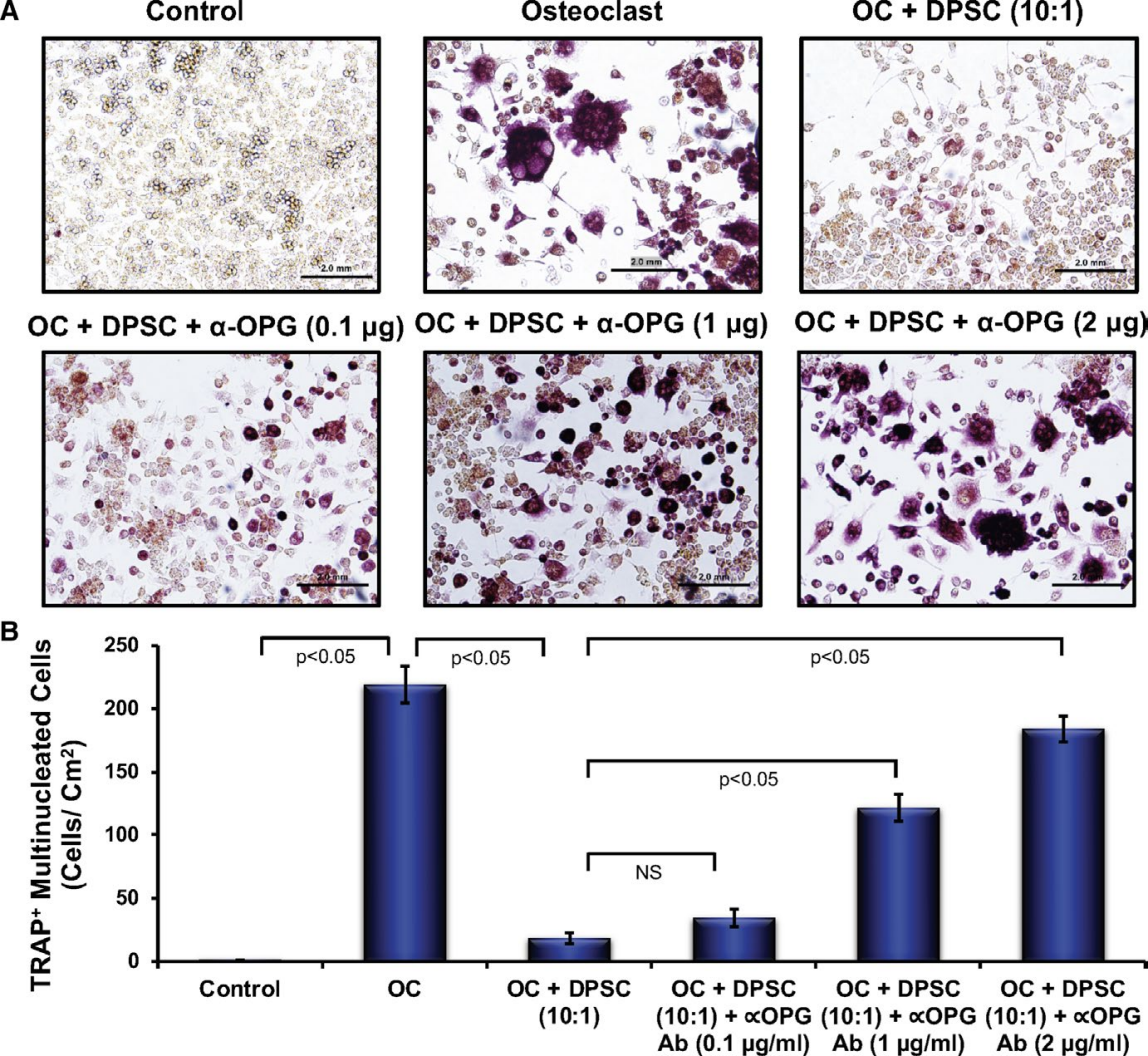
Anti‐OPG Ab‐mediated neutralization of secretory OPG to restore OC differentiation. A) TRAP staining images of RAW 264.7 cells at day 6 of OC differentiation are shown in the absence or presence of DPSC (10:1) with or without anti‐OPG antibody (0.1, 1 and 2 µg/mL). Scale bar = 2 mm. B) The number of TRAP‐positive multinucleated OCs is shown graphically in the absence or presence of DPSC and with or without anti‐OPG antibody (0.1, 1 and 2 µg/mL) during osteoclast differentiation

To determine whether DPSC‐mediated inhibition of osteoclast differentiation of RAW 264.7 cells was through OPG, we induced osteoclast differentiation in the absence or presence of DPSC or DPSC plus anti‐OPG Ab in a Transwell culture system. TRAP staining revealed that the presence of DPSC reduced osteoclast differentiation and addition of anti‐OPG Ab in the culture along with DPSC significantly restored the osteoclast differentiation in a dose‐dependent manner (Figure 8). These results confirmed that OPG‐mediated inhibition of osteoclast differentiation by DPSC in part.

## 4 DISCUSSION

Osteoclast differentiation of myeloid cells plays critical role in various bone‐related diseases such as arthritis, osteoporosis and Paget's bone disease ^2^, ^8^; hence, it is important to limit uncontrolled osteoclast differentiation to prevent excessive bone resorption in bone‐related diseases. Bone‐related diseases are often associated with compromised lifestyle, which causes significant health and socioeconomic burdens. Even though various pharmacological agents temporarily suppress joint erosion, regeneration of the deformed bones is quite limited and persists as a lifelong disability. Regenerative cell therapeutic approach would be a novel, and alternative strategy to treat arthritis and osteoporosis.^36^, ^37^, ^38^, ^39^ To bring these alternate regenerative approaches into practice, we need to better understand the insights of the differentiation of myeloid cells in to OCs and the potential mechanisms of adult stem cell–mediated regulation of various aspects osteoclast differentiation and functions both at the cellular and molecular levels.

To investigate the potential use of adult stem cells, we have isolated and expanded DPSCs from the third molar teeth using standard procedures following our earlier established protocol.^22^ These cells are mesenchymal in nature and express the cell surface markers CD73, CD90 and CD105, but do not express markers (CD34, CD133) of haematopoietic lineage cells and retain their ability to differentiate into multiple lineages including osteoblastic lineage.^22^ Given the inherent multipotency and immunomodulatory effects of DPSCs,^24^ we considered that these cells could be a new source for treating bone‐related diseases. As OCs play an important role in bone resorption and joint destruction in bone‐related diseases, we sought to investigate the possible effect of DPSC treatment on osteoclastogenesis because it is still remained unclear how DPSCs effect on myeloid cells. DPSCs are shown to exert trophic effects on various cells and are considered to be a potential therapeutic avenue for a number of musculoskeletal and autoimmune degenerative diseases.^40^

In our current study, we focused on generating OCs using established murine myeloid cells, RAW 264.7 cells with M‐CSF and sRANKL for osteoclast differentiation using earlier documented methods.^33^, ^41^, ^42^ After 6 days of differentiation, we observed multinucleated tartrate‐resistant acid phosphatase (TRAP)–positive cells confirming our ability in generating OCs. TRAP is a widely used osteoclast marker, which is known to be localized in the transcytotic vesicles of resorbing OCs that destroy collagen by producing reactive oxygen species.^43^ Our interesting finding of significant reduction in osteoclast differentiation process after co‐culturing with DPSCs suggest that the ability of DPSCs in inhibiting osteoclast differentiation process. We next investigated how DPSC inhibited osteoclast differentiation process. We first tested whether DPSC has any effect on osteoclast‐related molecules such as NFATc1, cathepsin K, RANK, TRAP and MMP9, which are critically involved in osteoclast maturation and resorption process. Specifically, NFATc1 is a transcription factor and a master regulator of RANKL‐induced osteoclast differentiation.^44^ Osteoclast‐specific genes, such as TRAP^45^ and cathepsin K,^46^ are directly regulated by NFATc1, indicating the significance of the NFATc1 in osteoclastogenesis. The induction and activation of NFATc1 integrate RANKL signalling in terminal differentiation of OCs.^45^ In addition, high levels of both the cathepsin K and MMP‐9 (gelatinase B) expression in osteoclast play central role in the bone resorption process.^47^, ^48^, ^49^ Our results of inhibiting OC differentiation after co‐culture with DPSCs were associated with the reduced expression of NFATc1, TRAP, cathepsin K and MMP9 confirming DPSC’s ability to suppress the terminal differentiation of osteoclast precursors to mature OCs, which is in consistent with the previous related findings.^50^

We next sought to find the mechanisms by which DPSCs inhibit osteoclast differentiation. Our findings suggest that DPSC‐mediated inhibition of osteoclast differentiation is mediated through at least two different mechanisms. DPSCs are known to contribute in tissue regeneration in a paracrine fashion by secreting various factors.^25^ We found that DPSCs markedly suppressed osteoclast differentiation by the production of OPG, along with the induction of OPG in RAW 264.7 cells. Osteoprotegerin is known to be an inhibitory molecule for RANKL‐dependent osteoclast differentiation and function,^51^ and RANKL neutralization improved bone resorption in osteoporosis and rheumatoid arthritis.^37^, ^38^, ^39^ In addition, it was shown that OPG deficiency exhibited severe osteoporosis in mice because of excessive bone resorption by OCs.^52^, ^53^ Our findings demonstrated that one of the possible mechanisms of DPSC‐mediated inhibition of osteoclastogenesis was through the secretion of OPG. We further confirmed that by the addition of recombinant OPG during OC differentiation experiments in RAW 264.7 cells and found that OPG indeed inhibited induced OC differentiation.

Sustained inflammatory responses result in developing chronic inflammatory diseases such as rheumatoid arthritis that are often associated with cartilage and bone destruction.^9^, ^10^ Inflammatory cytokines that are abundant in the synovial fluid and synovium of arthritic patients induce RANKL expression on synovial fibroblasts resulting in increased RANKL signalling.^9^, ^10^ Osteoclastogenesis process could be controlled by limiting inflammatory responses in the arthritic synovium. Our data revealed that co‐culture of DPSCs with osteoclast precursor cells lead to reducing inflammatory molecular expression in RAW 264.7 cells and polarization of M2 phenotype‐associated molecules in the RAW 264.7 cells. Previous study demonstrated that tonsil‐derived mesenchymal stem cells blunted the RANK‐RANKL interaction between the osteoclast precursor cell line RAW 264.7 and Th17 cells via OPG activity.^54^ These findings are in alignment with the others showing strong immunosuppressive functions of MSCs and DPSCs caused by soluble mediators such as anti‐inflammatory cytokines,^55^ and based on this intriguing property, the mesenchymal nature of DPSCs might have potential for therapeutic application in bone‐related diseases.

Signalling mechanism of osteoclast differentiation is well elucidated. M‐CSF and RANKL stimulation leads to osteoclast differentiation of precursor cells. M‐CSF is known to stimulate cell survival signalling mainly by activating extracellular signal–regulated kinase (ERK) through growth factor receptor–bound protein 2 (Grb‐2) and thymoma viral proto‐oncogene 1 (popularly called as AKT) through phosphatidylinositol 3‐kinase (PI3K) pathway.^56^ Similarly, RANKL stimulation leads to the formation of the RANK‐TRAF6 complex, that leads to the activation of the AKT, NF‐кB and MAPK pathways, including c‐jun N‐terminal kinase (JNK) and p38.^18^, ^56^ However, the signalling cascade that DPSCs or mesenchymal stem cells influence in regulating osteoclast differentiation is not well established. Here in, we have demonstrated that the secretory products of DPSCs were able to suppress activation of AKT and further inhibit the differentiation in the presence of M‐CSF and RANKL during osteoclastogenesis in part possibly through the secretion of OPG. To further confirm the role of OPG, we have shown that the AKT activation was inhibited by the addition of recombinant OPG in the presence of osteoclast stimulants M‐CSF and RANKL. This observation also suggests that the inhibition of osteoclast differentiation by DPSC in part could be of OPG‐mediated.

## 5 CONCLUSION

We show that human DPSCs inhibit osteoclastogenesis by reducing expression of key OC markers, such as NFATc1, cathepsin K, TRAP, RANK and MMP‐9 in RAW 264.7 cells through constitutive secretion of OPG. OPG potentially blocks the RANK‐RANKL interaction, which is essential for the differentiation of OCs. Moreover, DPSCs reduced inflammatory signals of RAW 264.7 cells and polarized RAW 264.7 cells towards M2 phenotype resulting in inhibition of osteoclast differentiation. We further confirmed that the trend of OPG‐mediated inhibition of the activation of PI3K signalling pathway (AKT) during OC differentiation was similar to the DPSC‐mediated inhibition of OC differentiation. The possibility of OPG‐mediated inhibition of OC differentiation by DPSCs was partially confirmed by rescue experiment using anti‐OPG antibody. This study provides evidence of DPSC‐mediated inhibition of osteoclastogenesis mechanisms.

## CONFLICT OF INTEREST

The authors have no conflict of interest.

## AUTHOR CONTRIBUTIONS

**Suman Kanji:** Data curation‐Lead, Formal analysis‐Lead, Writing‐original draft‐Lead. **Ripon Sarkar:** Data curation‐Supporting. **Asmita Pramanik:** Data curation‐Supporting. **Sudhir Kshirsagar:** Data curation‐Supporting. **Carl Greene:** Data curation‐Supporting. **Hiranmoy Das:** Conceptualization‐Equal, Formal analysis‐Equal, Funding acquisition‐Lead, Investigation‐Equal, Project administration‐Equal, Supervision‐Lead, Writing‐review & editing‐Lead.

## Supporting information

Fig S1‐S7
