# Supplementary material for: Dental pulp–derived stem cells inhibit osteoclast differentiation by secreting osteoprotegerin and deactivating AKT signalling in myeloid cells
Source: J Cell Mol Med. 2021 Jan 28;25(5):2390–403. doi: 10.1111/jcmm.16071 (PMC7933945; doi:10.1111/jcmm.16071)
Supplement: Supplementary file 1 — Fig S1‐S7 [file JCMM-25-2390-s001.pdf]

Dental pulp-derived stem cells inhibit osteoclast differentiation by secreting osteoprotegerin and deactivating AKT signaling in myeloid cells

Suman Kanji, Ripon Sarkar, Asmita Pramanik, Sudhir Kshirsagar, Carl J Greene and Hiranmoy Das*

Department of Pharmaceutical Sciences, School of Pharmacy, Texas Tech University Health Sciences Center, Amarillo, Texas, 79106

Supplementary Fig. 1

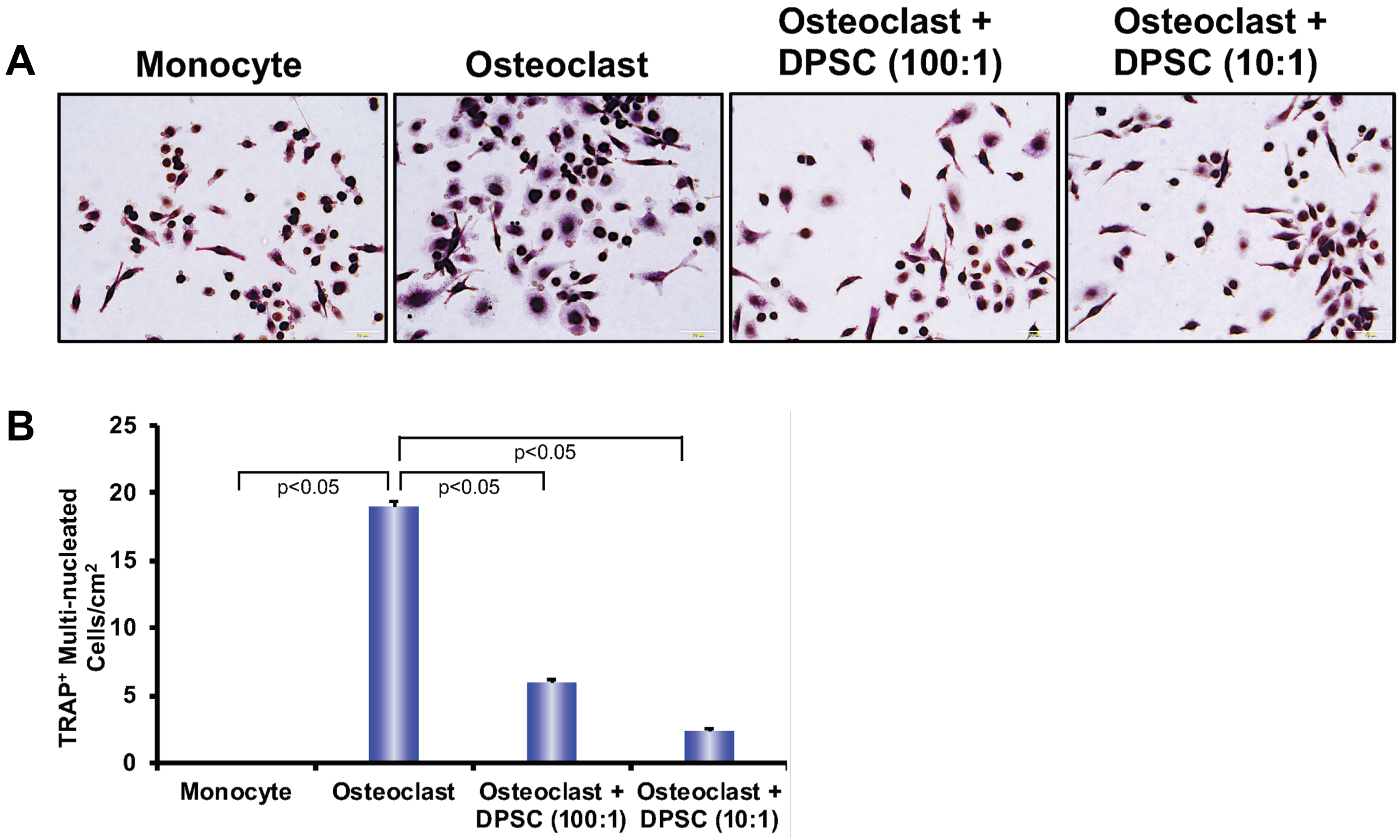

Supplementary Fig. 1. Dose-dependent inhibition of osteoclast differentiation using human primary monocytes. **A.** Images of induced differentiated primary human monocytes determined by TRAP staining at day 6 in the absence or presence of human DPSCs (contact-free co-culture) during osteoclast differentiation. **B.** The number of TRAP positive multinucleated osteoclasts present in each group shown graphically in absence or presence of human DPSCs (contact-free co-culture) during osteoclast differentiation.

Supplementary Fig. 2

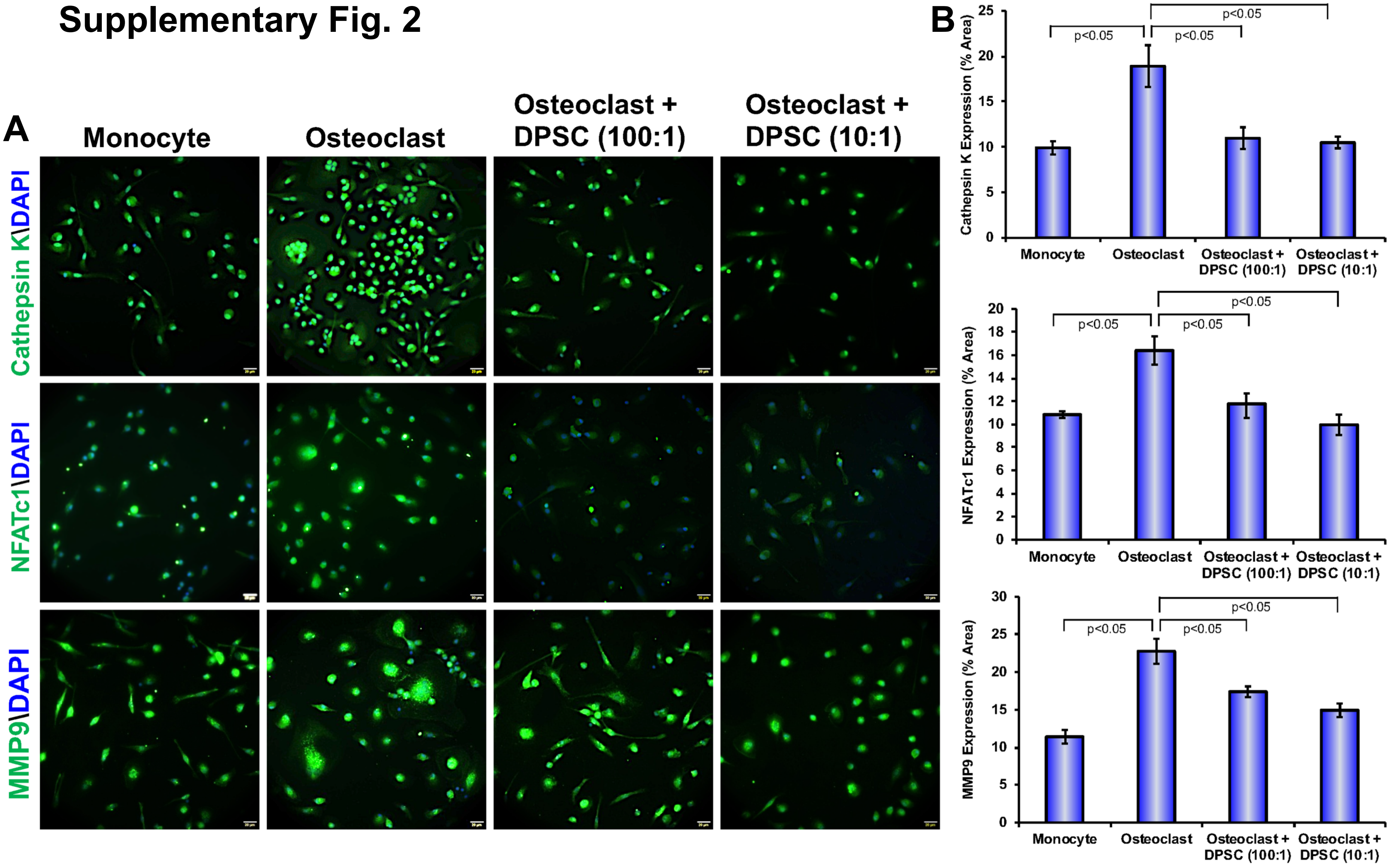

Supplementary Fig. 2. DPSCs inhibited osteoclast differentiation-related molecules in in primary human monocytes. **A.** Immunocytochemical staining images of osteoclast differentiation determined for cathepsin K, NFATc1, or MMP9 molecules at day 6 in the absence or presence of human DPSCs (contact-free co-culture) during osteoclast differentiation of primary human monocytes. **B.** Amount of cathepsin K, NFATc1, and MMP9 stains were quantified for each sample shown graphically in absence or presence of human DPSCs (contact-free co-culture) during osteoclast differentiation. All values are represented as mean ± SEM of 5 to 6 samples from one of the two experiments (from two independent donors).

Supplementary Fig. 3

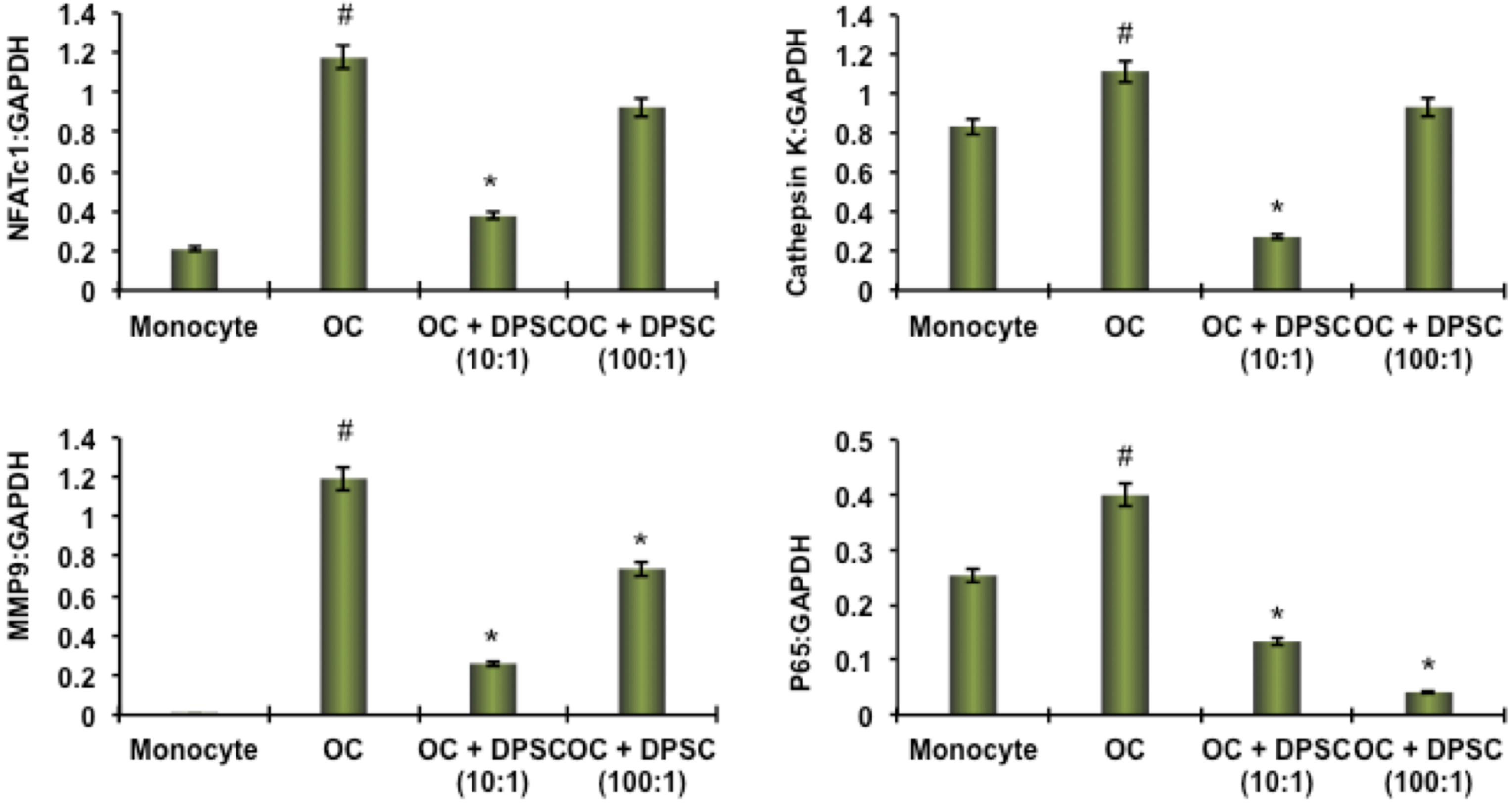

Supplementary Fig. 3. Bar graphs show the quantification of western blot data of NFATc1, Cathepsin K, MMP9 and P65 of Fig. 1D. Hashtag (#) indicates a statistical significance ($p<0.05$) when compared to monocytes, and star (*) indicates a statistical significance ($p<0.05$) when compared to osteoclasts (OC).

Supplementary Fig. 4

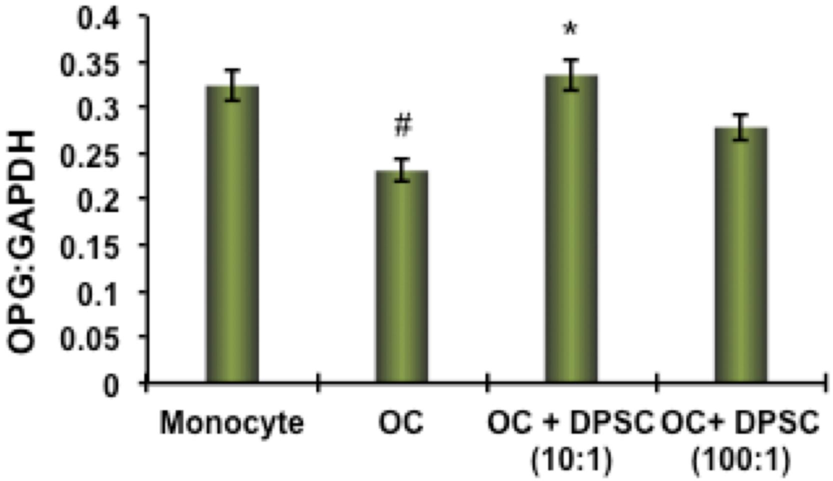

Supplementary Fig. 4. Bar graphs show the quantification of western blot data of OPG of Fig. 4B. Hashtag (#) indicates a statistical significance ($p<0.05$) when compared to monocytes, and star (*) indicates a statistical significance ($p<0.05$) when compared to osteoclasts (OC).

Supplementary Fig. 5

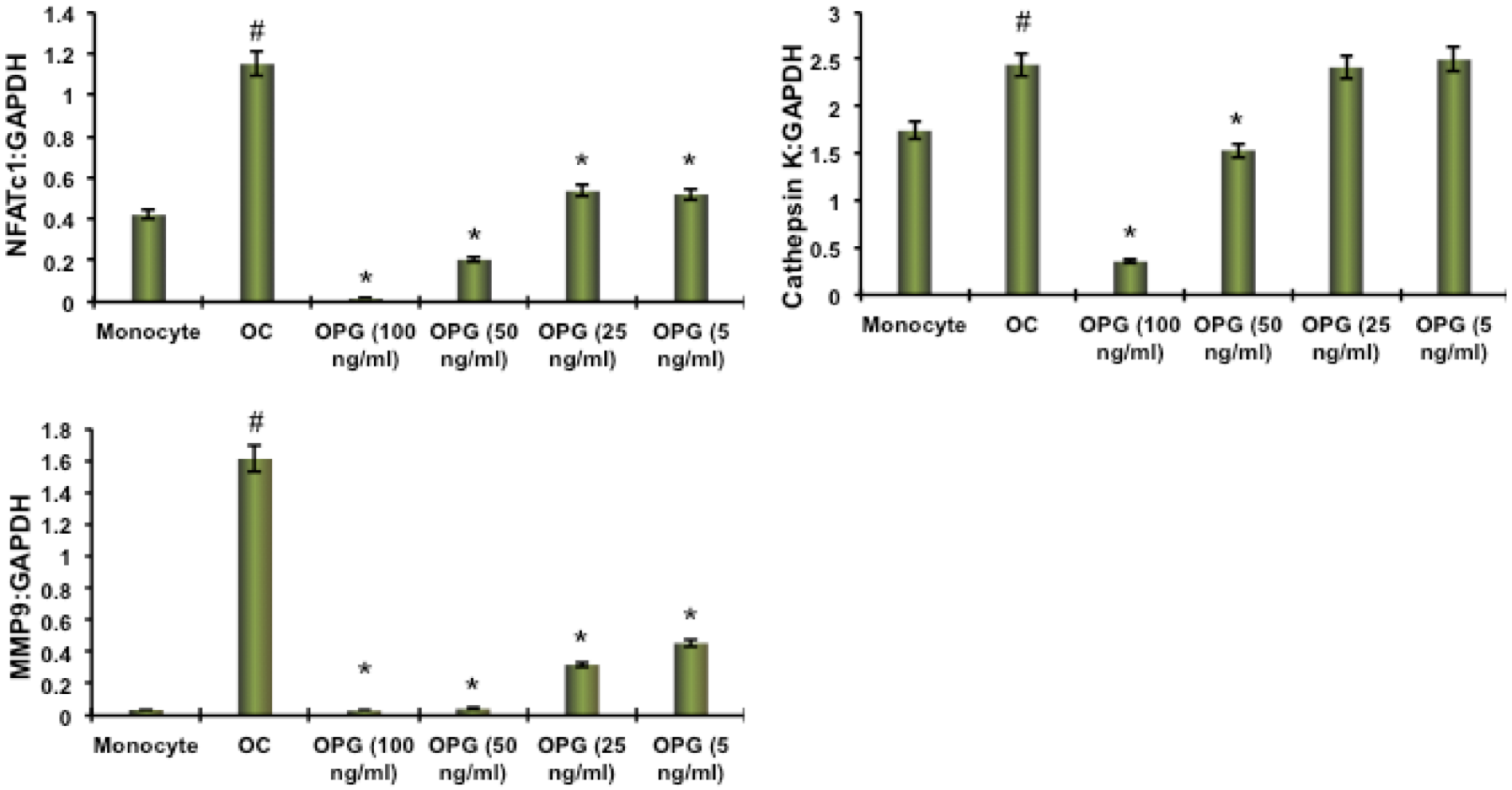

Supplementary Fig. 5. Bar graphs show the quantification of western blot data of NFATc1, Cathepsin K, MMP9 of Fig. 6B. Hashtag (#) indicates a statistical significance ($p<0.05$) when compared to monocytes, and star (*) indicates a statistical significance ($p<0.05$) when compared to osteoclasts (OC).

Supplementary Fig. 6

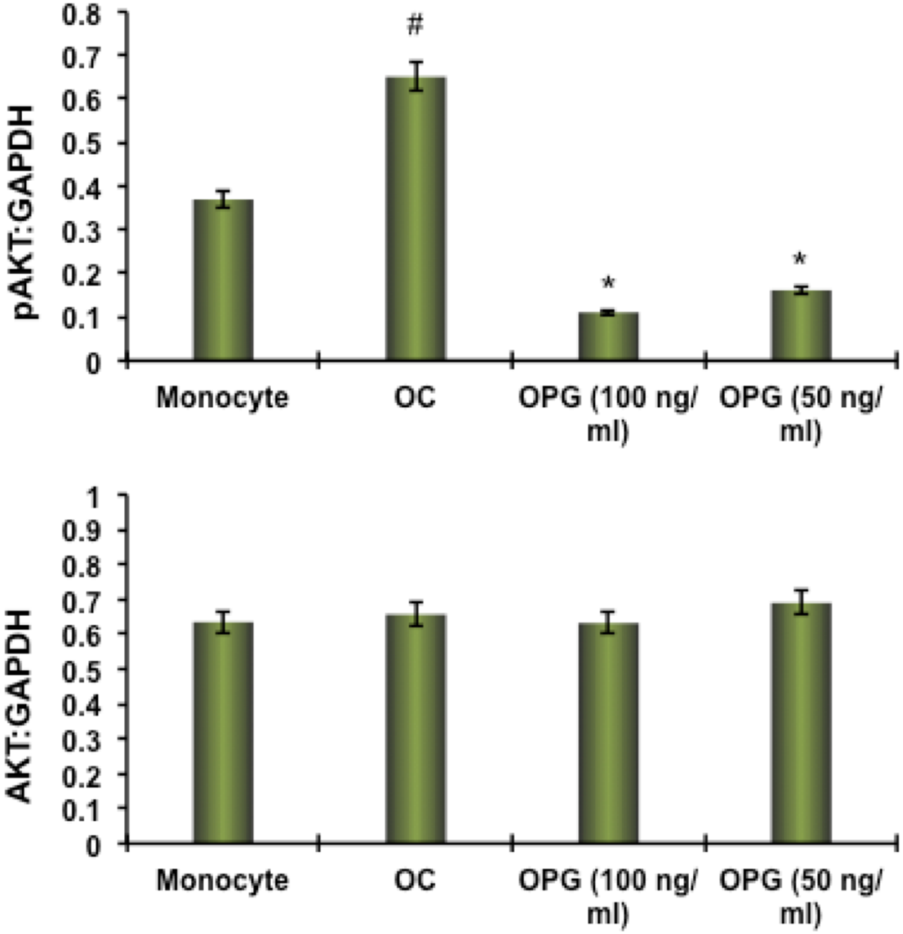

Supplementary Fig. 6. Bar graphs show the quantification of western blot data of pAKT and AKT of Fig. 7C. Hashtag (#) indicates a statistical significance ($p<0.05$) when compared to monocytes, and star (*) indicates a statistical significance ($p<0.05$) when compared to osteoclasts (OC).

Supplementary Fig. 7

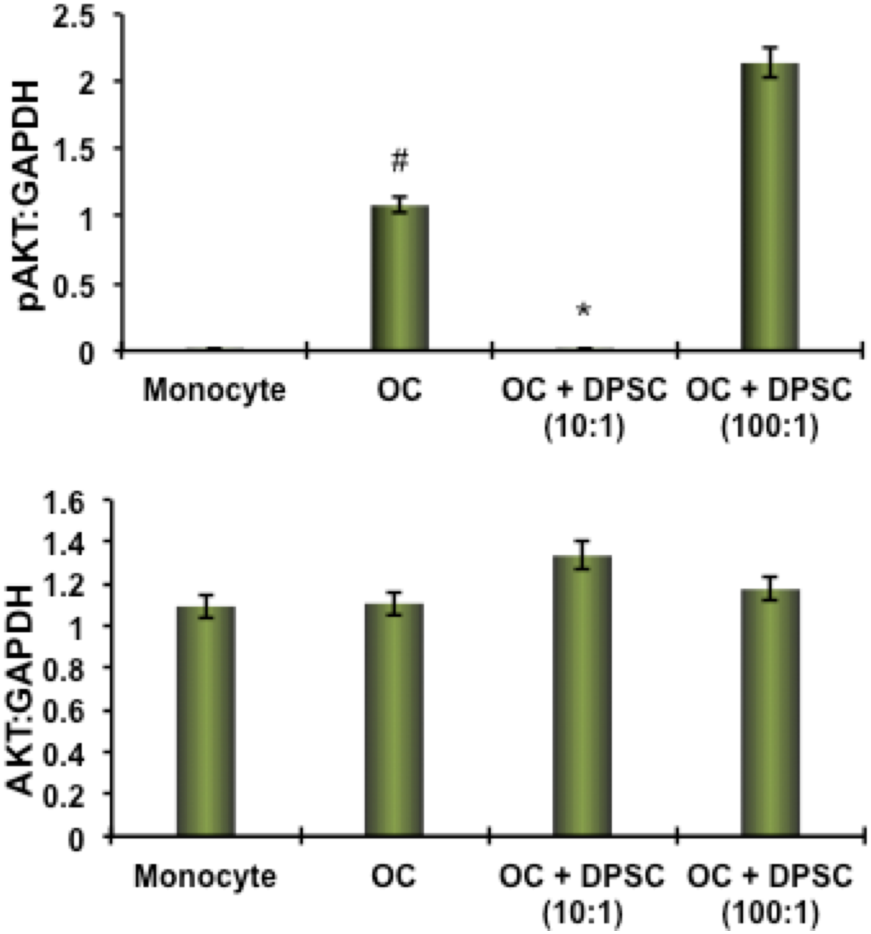

Supplementary Fig. 7. Bar graphs show the quantification of western blot data of pAKT and AKT of Fig. 7D. Hashtag (#) indicates a statistical significance ($p<0.05$) when compared to monocytes, and star (*) indicates a statistical significance ($p<0.05$) when compared to osteoclasts (OC).
